# Supplementary material for: Deep Kernel learning for reaction outcome prediction and optimization
Source: Commun Chem. 2024 Jun 14;7:136. doi: 10.1038/s42004-024-01219-x (PMC11178803; doi:10.1038/s42004-024-01219-x)
Supplement: Supplementary file 2 — Supporting Information [file 42004_2024_1219_MOESM2_ESM.pdf]

Supplementary Information for

Deep Kernel Learning for Reaction Outcome Prediction and Optimization

Sukriti Singh* and José Miguel Hernández-Lobato*

Department of Engineering, University of Cambridge, Cambridge, UK

sukriti243@gmail.com and jmh233@cam.ac.uk

	Table of Contents	Page
1.	Molecular representation	S3
	1.1 Molecular descriptors	S3
	1.2 Morgan fingerprints	S3
	1.3 Differential reaction fingerprints	S3
2.	Message passing graph neural networks	S4
3.	Implementation	S4
	3.1 DKL with learned representations	S4
	3.2 DKL with nonlearned representations	S5
4.	Performance metrics for the 80:20 data split	S7
5.	Out-of-sample prediction	S8
6.	Effect of fingerprint length	S8
7.	Effect of training set size	S9
8.	Feature analysis	S11

1. Molecular representations

We have tried various molecular representations as an input to the deep kernel learning (DKL) model. Here, we provide an overview of the representations used in this work.

1.1 Molecular descriptors

We use the molecular descriptors from the previous study (Ahneman et al. *Science* (2018)). These include global descriptors: molecular weight, EHOMO, ELUMO, hardness, ovality, electronegativity, surface area, molecular volume, and dipole moment. In addition, local descriptors to capture the atom-centric properties is also used. The examples include electrostatic charge, NMR shift, vibrational frequency and intensity. This results into a total of 120 descriptors for the reaction.

1.2 Morgan fingerprints

Molecular fingerprints are the most popularly used input representations in cheminformatics. They represent the molecule into a bit string format and are usually high-dimensional and sparse vectors. The basic idea behind computing the Morgan fingerprint involves few steps. It first assigns an identifier to each atom. Each atom's identifiers are sequentially updated based on the identifiers of the neighboring atoms. With each level of iteration, certain substructural features are added to an array. This is followed by hashing that converts the array to a bit vector representing the presence or absence of the substructures (Fig. S1).

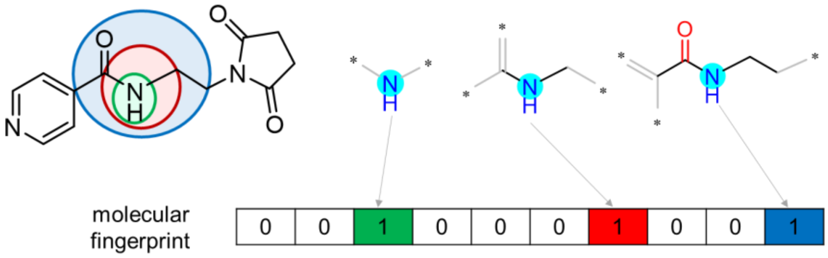

Fig. S1. A representative example of Morgan fingerprints.

1.3 Differential Reaction Fingerprints (DRFP)

The DRFP takes the reaction SMILES as an input and provides a binary fingerprint based on the symmetric difference of two sets containing molecular substructures listed on left and right sides of the reaction arrow. The length of the fingerprint vector is independent of the number of reaction components. It is implemented using the pypi package (drfp).

2. Message passing graph neural networks

Graph neural networks (GNN) are a class of neural networks that can operate directly on non-continuous input data like molecular graphs. In this work, we use a GNN inspired by the message passing neural network (MPNN) framework. The GNN captures the complex interactions between atoms and bonds by message-passing where each atom sends and receives messages based on the features of neighboring atoms and bonds. In this way, the local neighborhood information is aggregated in an iterative manner to obtain the global representation of the graph. The undirected graph with a set of node and edge features is usually taken as an input to GNN. Given the initial set of features, GNN has three main steps: (1) message-passing step, where a message is computed for each node using the node and edge features; (2) update step, where the node features are updated by aggregating the incoming messages from neighboring nodes; (3) readout step, where the node vectors are aggregated into a graph feature vector.

3. Implementation

In the following sections, we describe the implementation details of the DKL model with learned and nonlearned representations.

3.1 DKL with Learned Representations

The GNN component of the DKL model has a dimension of 64 for node representation vector and 512 for the graph/reaction representation vector. We considered three message passing steps and the number of set2set layers is also fixed to 3. No significant improvement in performance is observed with increasing the number of message passing steps. The reaction

representation obtained from GNN is passed to the feed-forward neural network (FFNN) with two fully-connected layers. It has a dimension of 256, followed by an output layer to obtain 128-dimensional embedding vector. We apply a dropout rate of 0.1 to the fully connected layers of FFNN. The embedding is passed as an input to the GP and Matérn52 without automatic relevance determination (ARD) is chosen as the base kernel. The model is trained for 400 epochs with a batch size of 128. The Adam optimizer with a learning rate of 0.001 is used to update all the parameters of the DKL model. A learning rate schedular is used to decay the initial learning rate to 0.0001 and 0.00001 for the last 100 epochs. All the calculations are implemented using PyTorch and GPyTorch libraries.

3.2 DKL with Nonlearned Representations

The input representation has a size of 120, 2048, and 2048 respectively for molecular descriptors, DRFP, and Morgan fingerprints. The fully-connected layer has a dimension of 100 with an output layer of size 50 for molecular descriptors. Similarly, it has a size of 512 followed by 256 for both DRFP and Morgan fingerprints. A dropout rate of 0.1 is applied to the NN layers. We use Matérn52 without ARD as the base kernel for GP. The model is trained for 400 epochs using the Adam optimizer with a learning rate of 0.001. The PyTorch and GPyTorch libraries are used for implementing the method. The nonlearned representations are also used to train a standard Gaussian process model. The size of input representation is same as above for molecular descriptors, DRFP, and Morgan fingerprints.

We have also investigated the model performance by summing the fingerprints of different reaction components instead of concatenation (Fig. S2). Each of the reaction component is first converted into 1024-bit fingerprint of radius 2. It is then passed through a linear layer to obtain an embedding of size 512. The reaction embedding is obtained by summing the embeddings of individual reaction components. It is then processed by a two-

layer feed-forward NN with a dimension of 256, followed by an output layer to obtain 128-dimensional embedding vector. This embedding is then passed as an input to the GP.

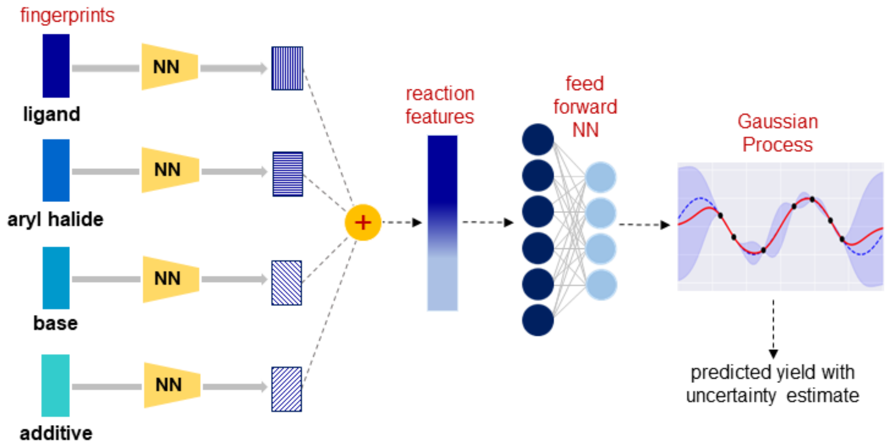

Fig. S2. A general representation of the DKL model architecture with nonlearned input representation.

The model performance is shown in Table S1.

Table S1. The Average Model Performance of DKL Method with Summation of Morgan Fingerprints of Reaction Components

Data split	RMSE	MAE	R^2
80:20	4.561±0.060	3.026±0.029	0.972±0.001
70:30	5.015±0.068	3.261±0.031	0.966±0.001
50:50	5.761±0.099	3.749±0.046	0.955±0.001
30:70	7.427±0.114	4.793±0.053	0.926±0.002
20:80	8.919±0.137	5.749±0.071	0.893±0.003
10:90	11.317±0.111	7.495±0.062	0.828±0.003
5:95	14.356±0.145	9.827±0.102	0.723±0.005

4. Performance metrics for the 80:20 data split

The model performance in terms of RMSE, MAE, and R^2, averaged over 10 independent runs is reported in Table S2.

Table S2. The Average Model Performance for the 80:20 Data Split for Various Methods

Representation	Method	RMSE	MAE	R^2
Graph	GNN	4.890±0.189	3.415±0.163	0.967±0.002
Graph	DKL	4.795±0.191	3.260±0.151	0.969±0.003
Molecular descriptor	GP	8.580±0.062	6.379±0.057	0.903±0.001

Molecular descriptor	DKL	4.866±0.066	3.352±0.038	0.969±0.001
Morgan FP	GP	6.388±0.055	4.716±0.046	0.945±0.001
Morgan FP	DKL	4.857±0.077	3.318±0.034	0.968±0.001
DRFP	GP	6.456±0.054	4.746±0.038	0.944±0.001
DRFP	DKL	5.151±0.143	3.531±0.078	0.964±0.002

We have performed paired t-test to analyze if the difference in mean between MorganFP_DKL and other models is statistically significant. The hypothesis is as follows:

Null hypothesis H_0: mean difference=0

Alternate hypothesis H_1: mean difference≠0

The p-value with a significance level (α) of 0.05 is used to evaluate if the difference in means is significant. It can be noted from Table S3 that the MorganFP-DKL model is significantly different from rest of the models, with GNN-DKL as an exception.

Table S3. p-values as Obtained from Paired t-Test

Sr. no.	Models	p-value
1	GNN and MorganFP-DKL	0.004
2	GNN-DKL and MorganFP-DKL	0.027
3	DRFP-GP and MorganFP-DKL	0.000
4	DRFP-DKL and MorganFP-DKL	0.001
5	MorganFP-GP and MorganFP-DKL	0.000

5. Out-of-sample prediction

We also evaluated the model performance on four out-of-sample splits based on the presence or absence of certain additives. The Morgan fingerprints are used with GP and DKL. In addition, the result with GNN-DKL is also presented (Table S4). The calculations are repeated 5 times for different random seeds and an average RMSE is reported in Table S4.

Table S4. Model Performance in terms of RMSE on Out-of-Sample Splits

	test1	test2	test3	test4
MorganFP-GP	9.771±0.000	11.054±0.000	15.240±0.000	18.761±0.000
MorganFP-DKL	9.629±0.164	11.492±0.193	16.123±0.144	19.300±0.211
GNN-DKL	9.935±0.768	11.310±0.989	16.755±1.282	19.097±1.057

6. Effect of fingerprint length

Morgan fingerprints for each reactant is computed as a 256-bit vector with radius 2. The reaction representation is a concatenation of fingerprints for individual reaction components, giving a 1024-dimensional bit vector. Also, we use DRFP with 1024 bits for comparison. The results are shown in Table S5.

Table S5. Model Performance with Morgan Fingerprints and DRFP of Length 1024

DKL with Morgan fingerprints			
Data split	R^2	RMSE	MAE
80:20	0.965±0.001	5.069±0.106	3.433±0.052
70:30	0.963±0.001	5.233±0.069	3.512±0.035
50:50	0.952±0.001	6.000±0.074	4.063±0.039
30:70	0.922±0.001	7.634±0.075	5.102±0.047
20:80	0.892±0.002	8.994±0.082	6.057±0.044
10:90	0.838±0.003	10.984±0.084	7.622±0.068
5:95	0.762±0.006	13.304±0.157	9.484±0.105
DKL with DRFP			
Data split	R^2	RMSE	MAE
80:20	0.968±0.001	4.902±0.101	3.370±0.047
70:30	0.960±0.001	5.426±0.046	3.639±0.027
50:50	0.949±0.001	6.177±0.063	4.133±0.038
30:70	0.917±0.001	7.853±0.075	5.251±0.050
20:80	0.886±0.002	9.214±0.092	6.200±0.054
10:90	0.831±0.003	11.229±0.102	7.851±0.080
5:95	0.749±0.004	13.667±0.118	9.766±0.094

7. Effect of training set size

The different train-test splits considered to investigate the effect of data size on model performance are: 70:30, 50:50, 30:70, 20:80, 10:90, 5:95. The model performance in terms of RMSE, MAE, and R^2, averaged over 10 independent runs is reported in Table S6.

Table S6. The Average Model Performance for Various Data Splits

GNN			
Data split	R^2	RMSE	MAE
70:30	0.965±0.002	5.112±0.140	3.595±0.091
50:50	0.952±0.003	5.973±0.155	4.174±0.082
30:70	0.920±0.004	7.654±0.288	5.298±0.125
20:80	0.888±0.008	9.077±0.349	6.235±0.201
10:90	0.805±0.020	11.960±0.600	8.424±0.420

5:95	0.719±0.021	14.450±0.503	10.719±0.441
DKL with molecular graph			
Data split	R^2	RMSE	MAE
70:30	0.967±0.002	4.987±0.109	3.406±0.078
50:50	0.951±0.004	5.995±0.241	3.995±0.108
30:70	0.925±0.010	7.282±0.491	4.847±0.230
20:80	0.900±0.010	8.500±0.518	5.646±0.271
10:90	0.831±0.015	11.211±0.478	7.534±0.289
5:95	0.718±0.030	14.454±0.763	10.152±0.585
GP with Molecular descriptors			
Data split	R^2	RMSE	MAE
70:30	0.893±0.002	9.001±0.074	6.713±0.047
50:50	0.870±0.002	9.845±0.064	7.406±0.034
30:70	0.829±0.001	11.328±0.031	8.557±0.027
20:80	0.793±0.002	12.447±0.064	9.464±0.052
10:90	0.716±0.004	14.542±0.105	11.234±0.069
5:95	0.572±0.061	17.548±1.038	13.918±0.992
DKL with Molecular descriptors			
Data split	R^2	RMSE	MAE
70:30	0.964±0.001	5.211±0.081	3.589±0.041
50:50	0.947±0.002	6.264±0.110	4.207±0.058
30:70	0.913±0.002	8.050±0.105	5.331±0.047
20:80	0.884±0.003	9.303±0.118	6.240±0.077
10:90	0.810±0.004	11.903±0.136	8.179±0.105
5:95	0.697±0.006	15.007±0.147	10.681±0.082
GP with Morgan fingerprints			
Data split	R^2	RMSE	MAE
70:30	0.935±0.001	6.920±0.043	5.033±0.036
50:50	0.917±0.001	7.849±0.059	5.726±0.041
30:70	0.879±0.001	9.508±0.059	7.007±0.029
20:80	0.846±0.002	10.712±0.048	8.003±0.039
10:90	0.786±0.003	12.650±0.091	9.708±0.070
5:95	0.708±0.005	14.756±0.123	11.504±0.116
DKL with Morgan fingerprints			
Data split	R^2	RMSE	MAE
70:30	0.962±0.001	5.272±0.083	3.572±0.053
50:50	0.951±0.001	6.012±0.066	4.068±0.040
30:70	0.923±0.002	7.568±0.083	5.060±0.042
20:80	0.894±0.002	8.900±0.081	5.997±0.040
10:90	0.836±0.003	11.071±0.096	7.681±0.074
5:95	0.760±0.006	13.381±0.180	9.520±0.122
GP with DRFP			
Data split	R^2	RMSE	MAE
70:30	0.933±0.001	7.025±0.048	5.120±0.030
50:50	0.915±0.001	7.956±0.057	5.828±0.039
30:70	0.877±0.001	9.576±0.044	7.113±0.033
20:80	0.846±0.001	10.730±0.038	8.060±0.038
10:90	0.786±0.003	12.631±0.078	9.704±0.076

5:95	0.707±0.004	14.787±0.090	11.502±0.086
DKL with DRFP			
Data split	R^2	RMSE	MAE
70:30	0.958±0.001	5.5531±0.103	3.732±0.060
50:50	0.950±0.001	6.089±0.042	4.086±0.029
30:70	0.919±0.001	7.794±0.073	5.204±0.041
20:80	0.889±0.002	9.122±0.079	6.145±0.044
10:90	0.829±0.003	11.308±0.079	7.871±0.059
5:95	0.751±0.005	13.611±0.147	9.696±0.115

8. Feature analysis

In order to get an insight into the features learned by the DKL model, we extracted the output of the last layer of fully connected NN. The size of the feature embedding is 256. We trained the DKL model on 80% of the data, and 20% is used for this analysis. The embedding of the 20% samples as obtained from the trained DKL model is then processed using Uniform Manifold Approximation and Projection (UMAP). The 256-dimensional embedding is reduced to two components. Next, a k-means clustering is performed on these two components. The Morgan fingerprint for the reaction has a dimension of 2048. We first reduce the dimension to two using the UMAP, followed by k-means clustering. The optimal number of clusters are determined using the elbow method. It calculates the Within-Cluster-Sum of Squared Errors (WSS) for various clusters and selects the k for which the change in WSS error starts to decline. The elbow plot is shown in Fig. S3. The elbow point is noted with k=4 clusters. Additionally, we also performed silhouette analysis which determines the separation distance between the clusters. A silhouette score closer to +1 indicates that the samples are far from the neighboring clusters. A score closer to 0 indicates that the samples are closer to the decision boundary of two neighboring clusters. Whereas, a negative score indicates that the sample might belong to the wrong cluster. We obtained average silhouette score of 0.53, 0.53, and 0.49 respectively for 4, 5, and 6 number of clusters. Given the similar values of the silhouette score and the elbow plot, we choose the optimal number of clusters to be 4. A similar trend is observed on cluster analysis using Morgan fingerprints.

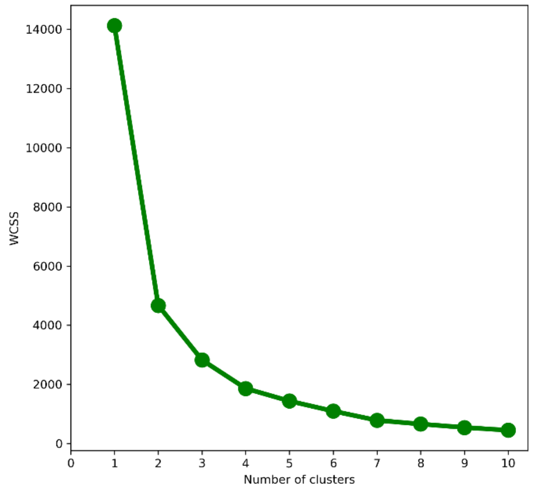

Fig. S3. Elbow plot to determine the optimal number of clusters.

Four distinct clusters are noticed in each case, details of which are presented in Table S8. It can be noted that for the Morgan fingerprint the clusters are formed based on ligand and base. The clusters 1, 2, and 4 have single base (**B2**, **B2**, **B2**) whereas cluster 3 has a combination of two bases (**B2** and **B3**). On the other hand, the clusters formed from the DKL features have all three bases present in all four clusters. We also investigated the median yield of each cluster. The median yield of the 20% data used for this analysis is 30.1. The median yield of each of the clusters is shown in Table S7. While the median yield of each cluster for Morgan fingerprints is closer to the overall median yield, we can see two distinct high and low yield clusters for DKL features. These are highlighted in blue.

Table S7. Structure of Ligands and Bases Used in this Study

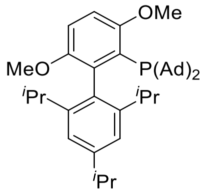 (Ad = adamantyl)	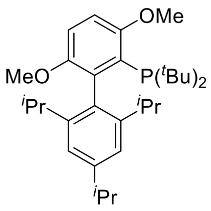	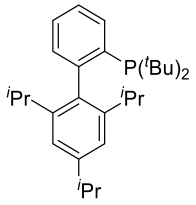	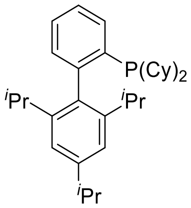
**L1**	**L2**	**L3**	**L4**
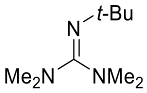	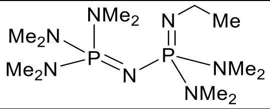	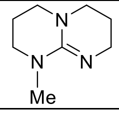	
**B1**	**B2**	**B3**	

Table S8. Identity of Ligand and Bases Present in Each Cluster Along with Median Yield (see Table S7 for identity of ligand and base)

	Feature	cluster1	cluster2	cluster3	cluster4
Median yield	Morgan fingerprint	38.4	21.0	29.0	27.1
	DKL Embedding	21.9	3.4	60.6	19.0
Identity of ligand and base	Morgan fingerprint	**B1**<br>**L1, L2, L3, L4**	**B2**<br>**L2, L3, L4**	**B2, B3**<br>**L1, L2**	**B3**<br>**L3, L4**
	DKL Embedding	**B1, B2, B3**<br>**L4**	**B1, B2, B3**<br>**L1, L2, L3, L4**	**B1, B2, B3**<br>**L1, L2, L3, L4**	**B1, B2, B3**<br>**L1, L2, L3, L4**

The distribution of yield of each cluster for DKL features and Morgan fingerprints is shown in Figs. S4 and S5 respectively.

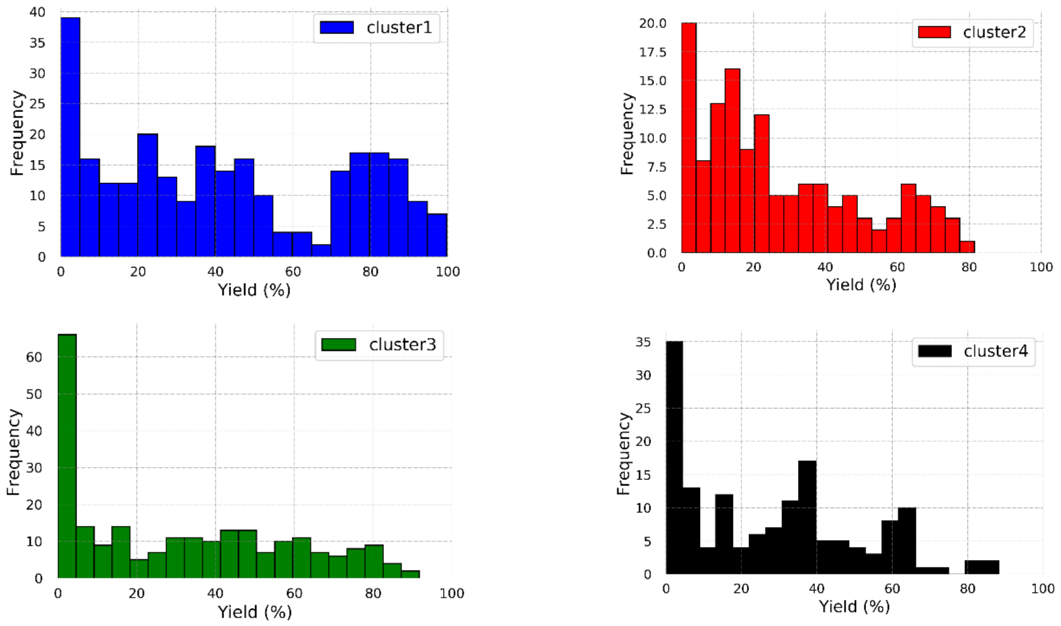

Fig. S4. Distribution of yields in different clusters as obtained from the Morgan fingerprints. The colors correspond to the clusters shown in Fig. 5b.

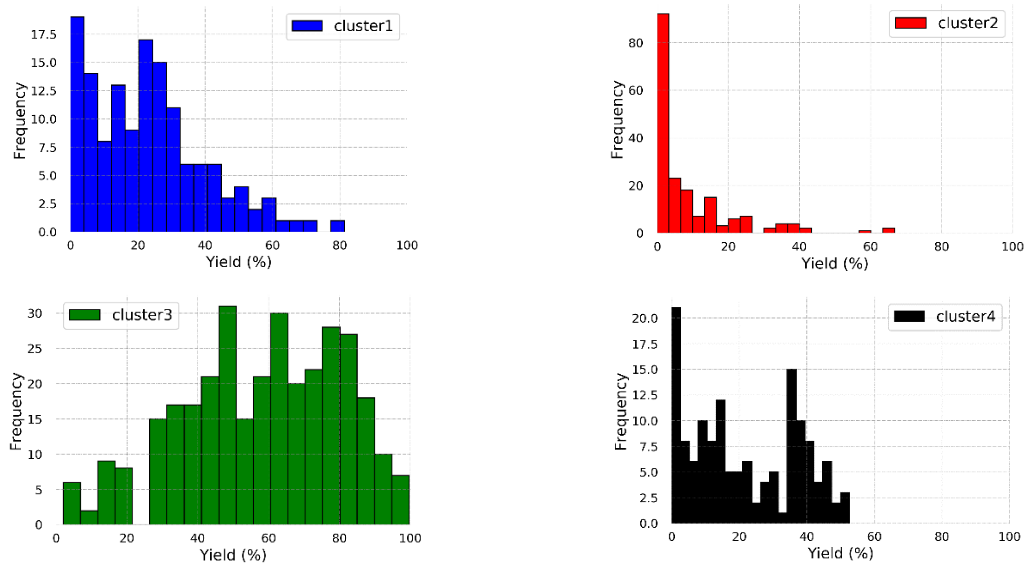

Fig. S5. Distribution of yields in different clusters as obtained from the embeddings of the DKL model. The colors correspond to the clusters shown in Fig. 5a.
